# Therapeutic Potential of Exercise-Induced SPARC in Bone Health?

**DOI:** 10.3390/biomedicines13040945

**Published:** 2025-04-12

**Authors:** Abdelaziz Ghanemi, Mayumi Yoshioka, Jonny St-Amand

**Affiliations:** 1 Functional Genomics Laboratory, Endocrinology and Nephrology Axis, CHU de Québec-Université Laval Research Center, Quebec, QC G1V 4G2, Canada; abdelaziz.ghanemi.1@ulaval.ca (A.G.); mayumi.yoshioka@crchudequebec.ulaval.ca (M.Y.); 2 Faculty of Pharmacy, Laval University, Quebec, QC G1V 0A6, Canada; 3 Université Laval’s Research Centre: The Tissue Engineering Laboratory (LOEX), Regenerative Medicine Division, CHU de Québec-Université Laval Research Center, Quebec, QC G1J 1Z4, Canada; 4 Department of Molecular Medicine, Faculty of Medicine, Laval University, Quebec, QC G1V 0A6, Canada

**Keywords:** secreted protein acidic and rich in cysteine (SPARC), bone, therapy

## Abstract

Exploring biological properties leading to potential pharmacological applications has been a fruitful approach in biomedical research. Secreted protein acidic and rich in cysteine (SPARC) is an exercise-induced glycoprotein known for its functions at different cellular and molecular levels. Among the properties it has, its calcium and collagen binding patterns along with other biochemical, metabolic, and structural effects represent a starting point towards developing therapeutic options based on SPARC properties for bones in pathological, preventive, and regenerative contexts. Such properties can be explored in conditions including bone fractures or requiring bone regenerative adjuvants. In addition, these properties can also be applied in basic research such as building an environment more suitable for cellular proliferation or optimizing in vitro conditions.

## 1. Exploring Molecular Properties Towards Therapeutic Applications

The modern challenge facing medical and therapeutic advances in certain areas is the lack of a mechanistic understanding of underlying diverse pathologies and health problems. This challenge also limits the development of therapeutic options. Therefore, uncovering molecular pathways would open up new doors towards identifying novel therapeutic targets and developing specific pharmacological tools. Studying biological, chemical, and biochemical patterns related to bone development, metabolism, remodeling, and structure, in various physiological and pathological contexts, allows us to pinpoint related biomolecules with possible therapeutic effects.

Understanding the roles of related specific proteins or factors, such as those impacting bone development and strength, will help us towards creating promising therapeutic approaches. Within this context, both collagen and calcium are key constituents within bones [1,2,3] that interact with secreted protein acidic and rich in cysteine (SPARC), that we focus on in this Viewpoint. SPARC has various properties exploitable for bone-related therapies. Therefore, this piece of writing describes the key properties that would allow for a therapeutic use of SPARC to increase bone health or reverse pathological statuses/conditions in addition to improving the bone bioenvironment towards healthier homeostasis. 

It is worth pointing out that SPARC has been reported to be associated with conditions such liver injury [4] and obesity [5]. Our hypothesis is that such SPARC overexpression does not necessarily mean that SPARC induced the disease or that it is implicated in its pathogenesis but that SPARC expression is rather feedback aiming to re-balance the homeostasis and reverse negative impacts via the various SPARC properties. These properties might not lead to an impact within a pathological environment probably due to a SPARC “Resistance” or insufficient expression of SPARC. Therefore, exploring how we can use SPARC properties to overcome such challenges remains another objective. 

## 2. SPARC Effects with a Focus on Bones Benefits 

SPARC, initially named osteonectin [6,7], was discovered in bones. It is a protein expressed in various tissues and implicated in a variety of roles in bones, including bone remodeling [8], bone regeneration and formation [9], metabolism [8,10], tissue repair and healing [11,12], and even breast cancer bone metastasis inhibition [13]. SPARC has, in addition to the roles it plays at the cellular and molecular levels, important “chemical” properties that make it of a particular importance in bones. Indeed, SPARC has an affinity to both calcium [14,15], including to the extracellular matrix [16], and collagen [17,18]. With bones having a high calcium content, SPARC plays an important role in tissue adhesion and hardness compared to other soft tissues [6,19]. Moreover, SPARC deficiency leads to osteopenia [20], which reflects the crucial role that SPARC plays in bone development and structure.

These properties SPARC has of binding to collagen and calcium have been illustrated in a previous publication that proposed an explanation of the “bone mineral density protection paradox” in obese patients with chronic kidney disease [21]. It presented a possible explanation of why obese patients suffering from chronic kidney disease have bone protection while developing vascular calcification. Both consequences would be due to calcium complex depots enhanced by SPARC that are overexpressed during obesity [5]. On the other hand, this same publication points to a possible side effect of such SPARC properties due—most properly—to its affinity for calcium as well. However, such side effects of SPARC would be limited to specific pathological conditions. For instance, in our specific example of vascular calcification, for individuals with normal calcium levels, increased SPARC levels would not lead to vascular calcification seen in patients suffering from chronic kidney disease who have hypercalcemia. This is supported by the fact that SPARC is overexpressed with exercise and has been linked to positive outcomes, which led to the hypothesis linking exercise-induced SPARC to the benefits of exercise [22,23,24,25,26]. These observations presented SPARC as a homeostatic factor improving the endogenous environment towards optimized growth, development, recovery, and bone diseases-reversing conditions and even considered it to be an exercise-mimicking molecule.

## 3. Perspectives 

To conclude, SPARC properties, both chemical/biochemical (affinity to calcium and collagen) and biological (cell growth, tissue remodeling and regeneration, bioenvironment improvement, etc.), present SPARC as a potential therapeutic option for selected bone diseases. Indeed, conditions involving the loss of strength, density, regeneration, or development of bones, as well as the loss of biochemical homeostasis, could benefit from the abovementioned SPARC properties. Such effects can be obtained via a physical exercise-induced SPARC expression increase. However, since some individuals with bone conditions are not able to perform the required amount of physical activity due to pathologies, aging, or disabilities, the pharmacological option of external SPARC therapeutic administration (injection) comes as a promising therapeutic approach. As a therapeutic option, it has already been shown that the injection of recombinant SPARC in mice is bioactive [24], reflecting the feasibility of SPARC injection for achieving therapeutic goals. 

It is worth emphasizing that due to SPARC properties (such as its affinity to both calcium and collagen), particular attention (pharmacovigilance) should be paid to patients who have pathologies or conditions that could create an endogenous environment interacting with SPARC and leading to side effects. Indeed, the potential interactions with other treatments or conditions could remain an important limitation. For illustration, diseases/drugs causing variations in calcium levels (hyperparathyroidism [27,28], vitamin D intake [29]) and collagen expression (collagenopathies [30]) could either limit/reduce the benefits of SPARC on bones or lead to side effects such as vascular calcification. In such cases, the mitigation strategies for these side effects would be to have strict control of SPARC-injected concentrations.

Moreover, we expect that once a SPARC receptor(s) is identified, a better understanding of its pharmacodynamics properties will lead to a deeper molecular exploration of SPARC-related therapies. Finally, the application of SPARC’s ability to induce a biological environment would have its implications in research on regenerative medicine and even provide adjuvants for treatment aiming to speed up the healing of bone fractures. Such properties can also be applied within tissue engineering, for instance, by adding SPARC as an adjuvant or a “growth factor” to bone tissues.
